# Supplementary figures and images for: High-level carbapenem tolerance requires antibiotic-induced outer membrane modifications
Source: PLoS Pathog. 2022 Feb 7;18(2):e1010307. doi: 10.1371/journal.ppat.1010307 (PMC8853513; doi:10.1371/journal.ppat.1010307)

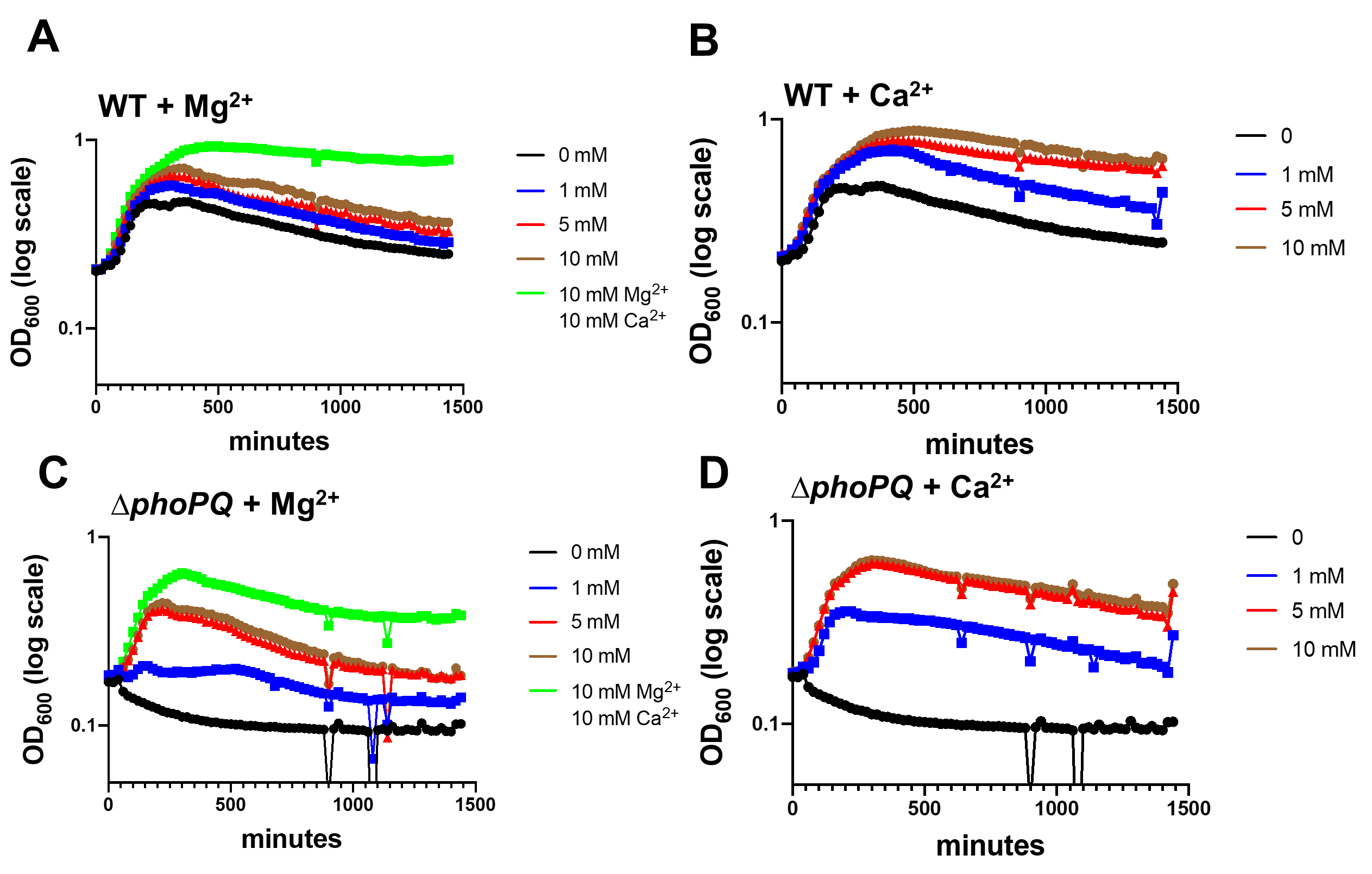

Supplement: S1 Fig — Wild type (WT) (A-B) or its ΔphoPQ derivative (C-D) were treated as described in Fig 1A with addition of the indicated concentrations of (A,C) MgSO4 (Mg2+) or (B,D) CaCl2 (Ca2+). Data represent the average of 3 replicates +/- standard deviation. (TIF) [file ppat.1010307.s001.tif]

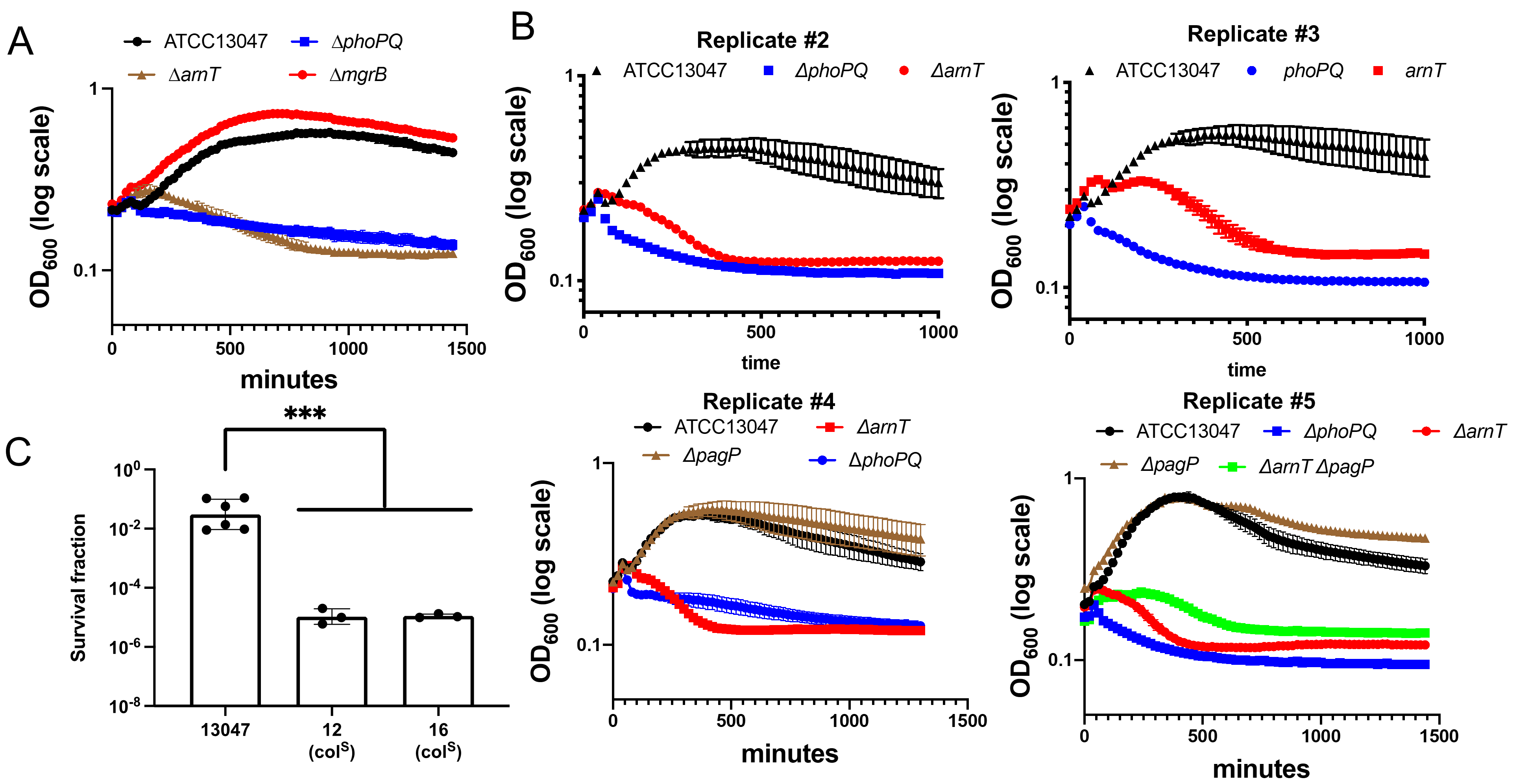

Supplement: S2 Fig — (A) An mgrB mutation promotes a moderate increase in mass increase during meropenem exposure. (B) Experiments were conducted as described in Fig 1A legend; each graph represents experiments conducted on a different day. In addition, data in each graph represent the average of 3 biological replicates +/- standard deviation. (C) Fraction of colistin-susceptible populations surviving after 24 hours of 10 μg/mL meropenem exposure. Statistical significance determined by one-way ANOVA of log transformed data, followed by Tukey’s correction for multiple comparisons (ns, not significant; ***, p ≤ 0.001). (TIF) [file ppat.1010307.s002.tif]

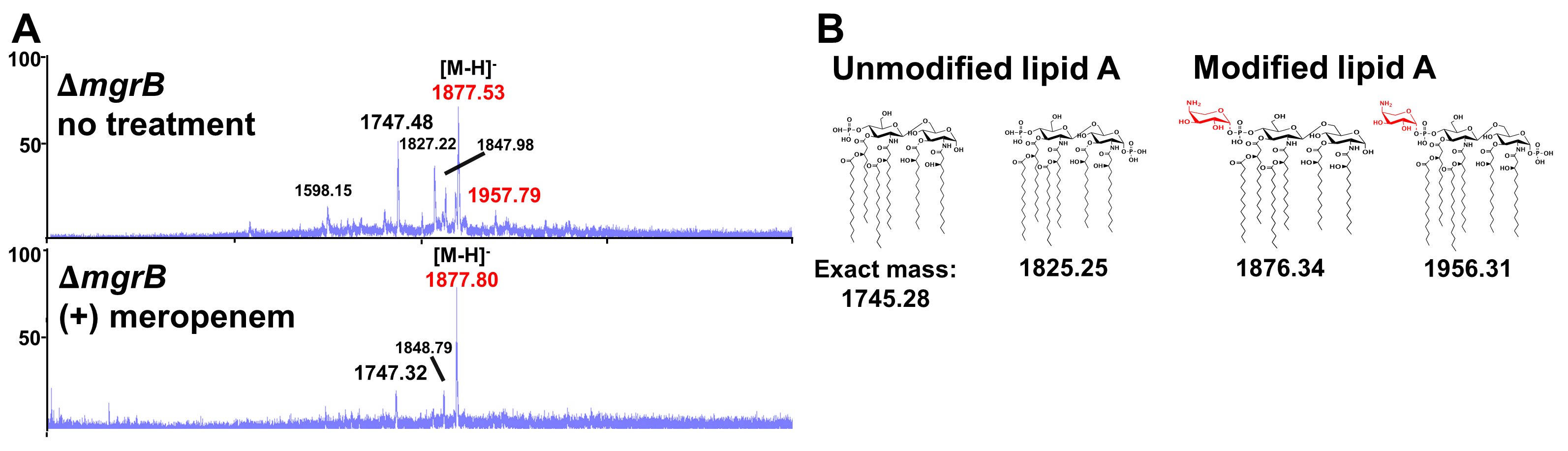

Supplement: S3 Fig — (A) MALDI-MS analysis of lipid A isolated from E. cloacae ΔmgrB. m/z corresponding with L-Ara4N modifications are illustrated in red. Each experiment was independently replicated three times, and one representative data set was reported. (B) Relevant lipid A chemical structures are shown. (TIF) [file ppat.1010307.s003.tif]

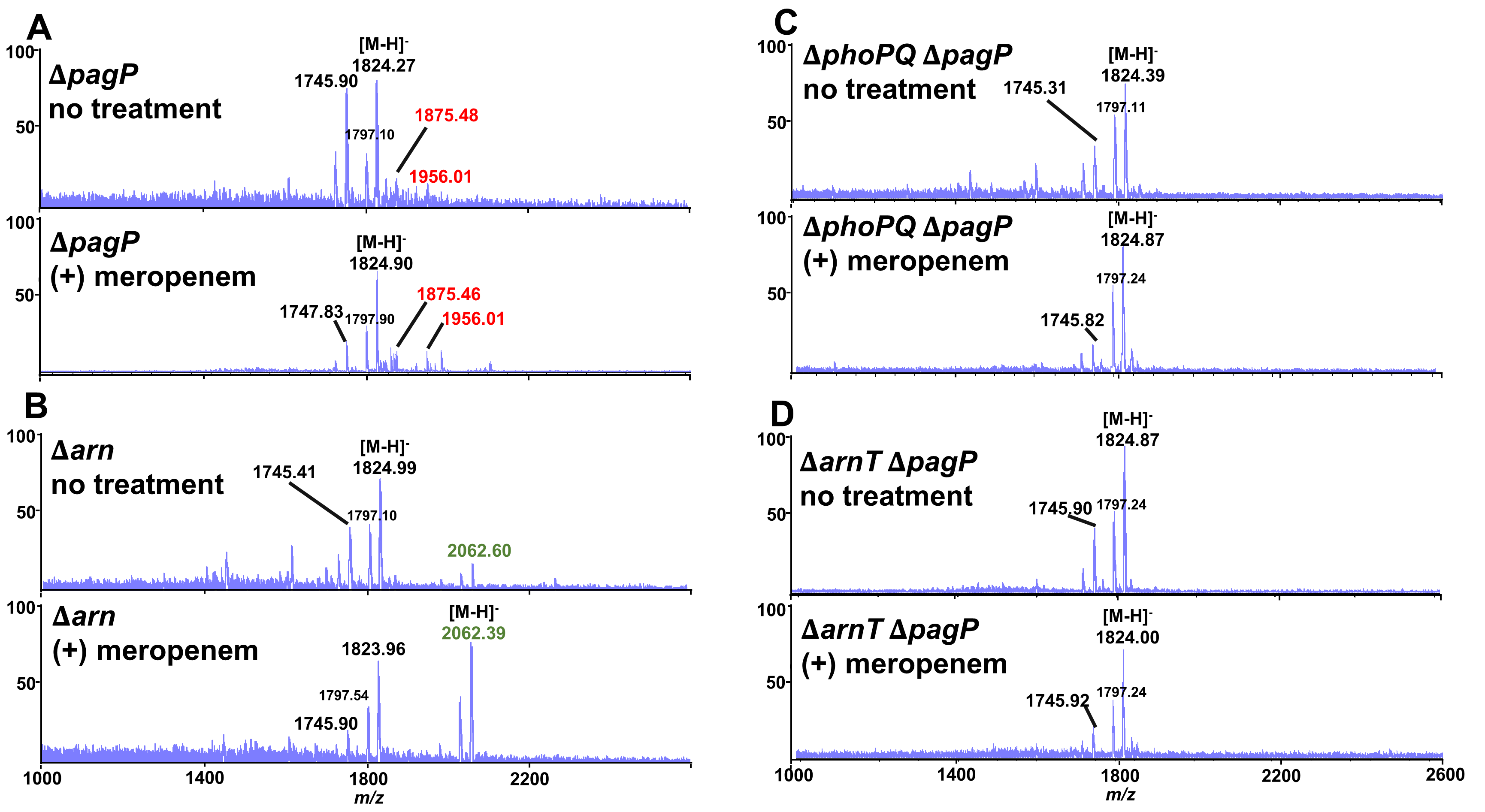

Supplement: S4 Fig — (A) MALDI-MS analysis of lipid A isolated from ΔpagP, (B) Δarn (full operon deletion), (C) ΔphoPQ ΔpagP and (D) ΔarnT ΔpagP. m/z corresponding with L-Ara4N modifications are illustrated in red, while structures with altered acyl chain patterns are illustrated in green. Each experiment was independently replicated three times, and one representative data set was reported. (TIF) [file ppat.1010307.s004.tif]

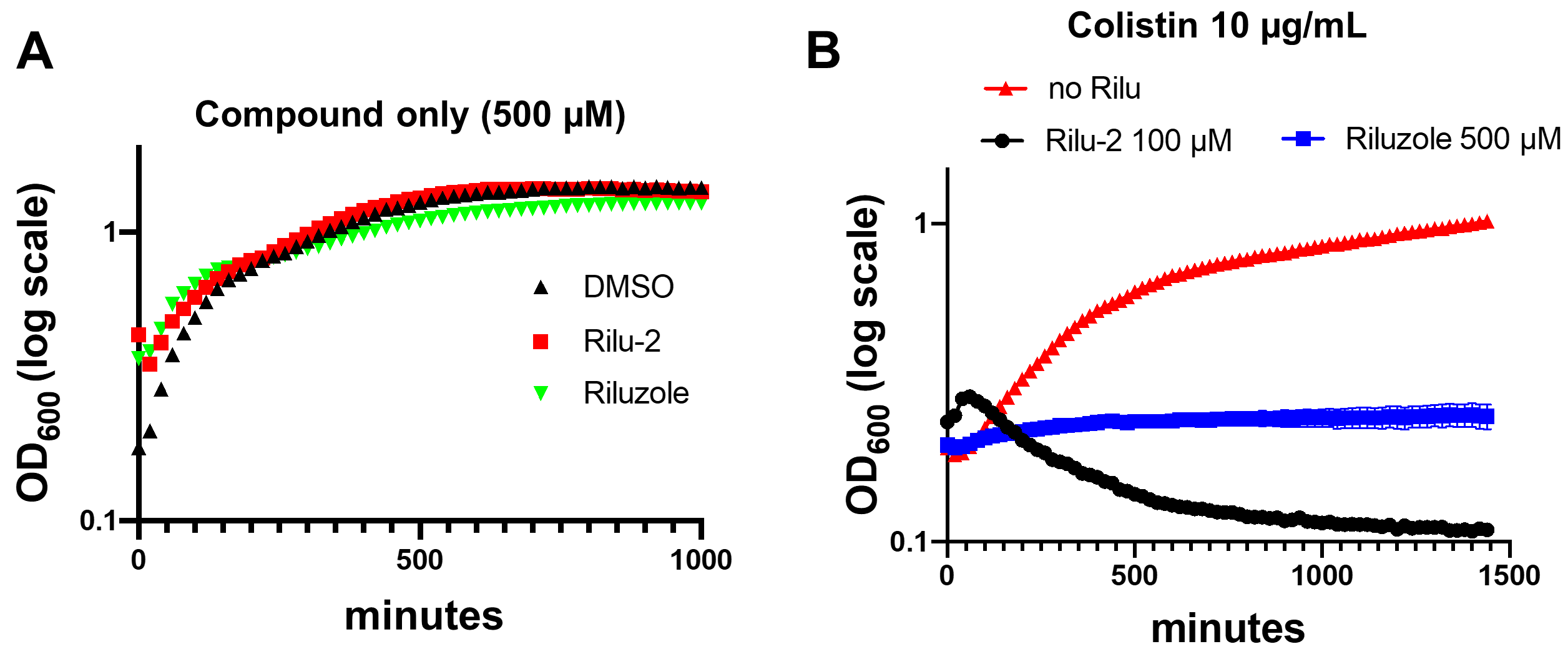

Supplement: S5 Fig — (A) Rilu compounds do not cause lysis, but (B) potentiate colistin mediate killing. Experiments were conducted as described in Fig 1A legend. Data represent the average of 3 replicates +/- standard deviation. (TIF) [file ppat.1010307.s005.tif]
