# Supplementary material for: High-level carbapenem tolerance requires antibiotic-induced outer membrane modifications
Source: PLoS Pathog. 2022 Feb 7;18(2):e1010307. doi: 10.1371/journal.ppat.1010307 (PMC8853513; doi:10.1371/journal.ppat.1010307)
Supplement: S1 Table — (DOCX) [file ppat.1010307.s006.docx]

S1 Table: Strains, plasmids and peptides used in this study

| **Strain or Plasmid** | **Antibiotic Resistance** | **Genotype or Plasmid Description** | **Reference** |
| --- | --- | --- | --- |
| **Strain** |  |  |  |
| *E. cloacae* strain ATCC 13047 | *Col^R^-hetero | Wild type | (1) |
| ATCC 13047 Δ*phoPQ_Ecl_* | Col^S^ | Δ*phoPQ_Ecl_* | (2) |
| ATCC 13047 Δ*phoPQ_Ecl_ /* pMMBKn::PhoPQ_Ecl_ | Col^R^-hetero,  Kn^R^ | Δ*phoPQ_Ecl_ /* pMMBKn::PhoPQ_Ecl_ | (2) |
| ATCC 13047 Δ*arnT_Ecl_* | Col^S^ | Δ*arnT_Ecl_* | (2) |
| ATCC 13047 Δ*mgrB_Ecl_* | Col^R^-hetero | Δ*mgrB_Ecl_* | This study |
| ATCC 13047 Δ*pagP_Ecl_* |  | Δ*pagP_Ecl_* | This study |
| ATCC 13047 Δ*phoPQ_Ecl_* Δ*pagP_Ecl_* | Col^S^ | Δ*phoPQ_Ecl_* Δ*pagP_Ecl_* | This study |
| ATCC 13047 Δ*arnT_Ecl_* Δ*pagP_Ecl_* | Col^S^ | Δ*arnT_Ecl_* Δ*pagP_Ecl_* | This study |
| *E. coli* W3110 | Col^S^ | Wild type | Genetic Stock Center (Yale) |
| *E. coli* WD101 | Col^R^ | W3110 constitutive *pmrA* mutant | (3) |
| *Klebsiella pneumoniae* 1084 | Col^S^, mero^S^ | Derivative of CDC AR isolate bank AR 0080, cured of its imipenemase | This study |
| *E. cloacae* 12 | Col^S^, mero intermediate^#^ | Clinical isolate | This study |
| *E. cloacae* 16 | Col^S^, mero^S $^ | Clinical isolate | This study |
| **Plasmids** |  |  |  |
| pMMB67EHKn:: | Kn^R^ | Empty vector | (4) |
| pMMBKn::PhoPQ_Ecl_ | Kn^R^ | The genetic coding sequence of *phoPQ_Ecl_* cloned into the KpnI and XbaI sites of pMMB67EHKn:: | (2) |
| *Col^R^-hetero denotes heteroresistance  ^#^meropenem MIC = 5 µg/mL  ^$^meropenem MIC = 0.125 µg/mL |  |  |  |

**References**

1. Guerin F, Isnard C, Sinel C, Morand M, Dhalluin A, Cattoir V, et al*.* Cluster-dependent colistin hetero-resistance in Enterobacter cloacae complex. J Antimicrob Chemother. 2016;71: 3058–3061.

2. Kang KN, Klein DR, Kazi MI, Guerin F, Cattoir V, Brodbelt JS, et al*.* Colistin heteroresistance in Enterobacter cloacae is regulated by PhoPQ-dependent 4-amino-4-deoxy-l-arabinose addition to lipid A. Mol Microbiol. 2019;111: 1604–1616.

3. Trent MS, Ribeiro AA, Doerrler WT, Lin S, Cotter RJ, Raetz CRH. Accumulation of a polyisoprene-linked amino sugar in polymyxin-resistant Salmonella typhimurium and Escherichia coli: structural characterization and transfer to lipid A in the periplasm. J Biol Chem*.* 2001;276: 43132-43144.

4. Boll JM, Crofts AA, Peters K, Cattoir V, Vollmer W, Davies BW, et al*.* A penicillin-binding protein inhibits selection of colistin-resistant, lipooligosaccharide-deficient Acinetobacter baumannii. Proc Natl Acad Sci U S A 2016;113, E6228-E6237.
