# Supplementary material for: High-level carbapenem tolerance requires antibiotic-induced outer membrane modifications
Source: PLoS Pathog. 2022 Feb 7;18(2):e1010307. doi: 10.1371/journal.ppat.1010307 (PMC8853513; doi:10.1371/journal.ppat.1010307)
Supplement: S2 Table — (DOCX) [file ppat.1010307.s007.docx]

S2 Table: Oligos used in this study

| TDP1532 | PagP1_wanner_fw2 | **GTGATCTTACGTAATAAATTTTTCTTCATTTTTCTGTTTATTTTTGCACAGTTAACGCTCTCTTCTGTGGCACAG**tgtgtaggctggagctgcttcg |
| --- | --- | --- |
| TDP1533 | PagP1_wanner_rev2 | **TTAAAACTGAAAACGCATCCAGGCGAAGTAAACGTTACCGTTGTTGTACGTACCGGGAATGTAGGTCATCTGGAA**catatgaatatcctccttag |
| TDP1767 | mgrB_fw_pTox | **ggcggggttttttcgttgatcacgtacgat**CGGTATTTGACCGCTTTGGTGTG |
| TDP1768 | mgrB_up_rev | **TCATCATTATTACTCGAGTGCGGCCGCATTA**TCCACACTCCGTCTTAACACCTG |
| TDP1769 | mgrB_down_fw | **TAATGCGGCCGCACTCGAGTAATAATGATGA**TGCGCAATCAATAAGTTTATTCCGTGGTAA |
| TDP1770 | mgrB_down_rev | **cctgtacaccatgtgcaccggttcgaagat**CCACCACTGGCGGATAAAGTC |
| TDP1771 | mgrB-flank_fw | CCTGTATGCAACAGGTGTTAAGACGG |
| TDP1772 | mgrB-flank_rev | CCATCTTCATGAGGTTGATTAAGCGAC |
| TC_120 | 1084 ΔphoP 1 | ggcggggttttttcgttgatcacgtacgatCATAAAACAGGCATGAATATACTCC |
| TC_344 | ∆phoPQ Rev 2 | GAAAAATCTCAACGAGGCTGTTCTCCCTAGCATTTTCGATAG |
| TC_345 | ∆phoPQ For 3 | CTAGGGAGAACAGCCTCGTTGAGATTTTTCACGGCGC |
| TC_127 | 1084 ΔphoQ 4 | cctgtacaccatgtgcaccggttcgaagatGTTCGGTCGGTAAAATATCC |
| TC_166 | phoPQ validation FOR | CCGTACAGCGTTCTGAAATCC |
| TC_167 | phoPQ validation REV | GCTCCGGTTGGTTAATCAGACC |
| TC_391 | phoP-FLAG (WT) | tttgggctagcgaattcgagctcggtacccAGGAGGATGCATATGCGCGTACTCGTGGTTGAGGATAATGCCCTGCTGCGTCACCACCTCAAAGTTCAGCTGCAGGAGCTGGGCCATCAGGTCGATGCGGCGGAAGATGCCAGGGAAGCGGACTACTATCTGGGCGAACATCTCCCGGATATCGCCATCGTCGATCTCGGCCTGCCGGATGAAGACGGTTTATCACTGATCCGCCGCTGGCGCAGCCACGACGTGTCGCTGCCGGTGCTGGTGCTGACCGCCCGCGAAGGATGGCAGGATAAAGTGGAAGTGCTGAGCGCCGGGGCGGATGATTACGTCACCAAGCCTTTCCATATTGAAGAGGTTGCCGCCCGCATGCAGGCGCTGCTGCGCCGTAACAGCGGCCTGGCCTCGCAGGTGATCTCCCTGCCGCCGTTCCAGGTCGACCTCTCCCGGCGCGAGCTGTCGGTGAATGACCAGCCGATCAAGCTGACCGCCTTTGAATACACCATTATGGAAACCCTGATCCGTAACCGCGGCAAAGTGGTCAGCAAAGATTCGCTGATGCTCCAGCTTTACCCGGATGCCGAACTGCGAGAAAGCCACACCATCGACGTGCTGATGGGTCGGCTGCGCAAGAAAATTCAGGCTGAATACCCACAGGACGTCATCACCACGGTGCGCGGCCAGGGCTATCTGTTCGAACTGCGCGGTGGCgactacaaagaccatgacggtgattataaagatcatgacatcgattacaaggatgacgatgacaaaTGAggggatcctctagagtcgacctgcaggcat |

*bold typeface = homology overhangs
